# Supplementary material for: Changes in corneal thickness after vitrectomy—Implications for glaucoma practice
Source: PLoS One. 2021 Apr 21;16(4):e0249945. doi: 10.1371/journal.pone.0249945 (PMC8059830; doi:10.1371/journal.pone.0249945)
Supplement: S1 Table — (PDF) [file pone.0249945.s001.pdf]

S1 Table. Comparison of various variables in affected eyes with and without pre-existing glaucoma

	Pre-existing glaucoma in affected Eye<br>N = 37	No pre-existing glaucoma in affected Eye<br>N= 90	P value
CCT in affected eye	570 ± 63.20	565 ± 84	0.7
ΔCCT	27 ± 8.2	39 ± 41.29	0.65
Presenting IOP<br>(mm Hg)	19 ± 9.77	31 ± 9.34	<0.0001
CDR	0.6 ± 0.24	0.3 ± 0.18	0.002
BCVA	1.3 ± 0.87	1.7 ± 0.68	0.01
Number of eyes requiring glaucoma surgery	3	9	0.7

IOP-intraocular pressure; CCT-central corneal thickness; CDR-cup disc ratio, BCVA-visual acuity (LogMar)
