# Supplementary material for: Changes in corneal thickness after vitrectomy—Implications for glaucoma practice
Source: PLoS One. 2021 Apr 21;16(4):e0249945. doi: 10.1371/journal.pone.0249945 (PMC8059830; doi:10.1371/journal.pone.0249945)
Supplement: Attachment — Submitted filename: Review Comments (13JAN21).docx [file pone.0249945.s002.docx]

**Changes in corneal thickness after vitrectomy -implications for glaucoma practice**

**Synopsis**

Patients referred to glaucoma services for possible secondary glaucoma after vitrectomy and who had undergone corneal pachymetry between July 2013 to June 2020, were included. The eye that developed elevated intraocular pressure (IOP) and diagnosed clinically as glaucoma after vitrectomy, was labelled as the “affected” eye. The contralateral eye of the patient with normal IOP and no history of vitrectomy, was labelled as the “control” eye. The difference in CCT in the affected eye and the contralateral control eye (ΔCCT) and CCT were compared.

**Overall Impression**

For the most part, the explanation of the methods is clear, including the plan for statistical analysis. However, the presentation of the results is confusing if the reader tries to relate it to the methods. The main confusion relates to the comparison of affected and contralateral eyes. It is possible that correct analyses were carried out and that the authors can clarify their presentation of results.

**Issues**

The authors state that the comparison of CCT between the control and affected eye was done using paired t-test and that the difference in CCT in the affected eye and the contralateral control eye (hitherto referred as ΔCCT) was being analyzed. This would be the correct analysis. However, it is not clear to me that they actually took the difference between affected and control eyes for each patient and analyzed the average difference as opposed to averaging the affected eye, averaging the control eyes and testing the difference in the means as if they were independent.

There are several reasons why I say this:

1. They state that if both eyes of the same patient had undergone vitrectomy, both eyes were labelled as affected. (In this case you would not be able to take the difference between control eyes and affected eyes.
2. They state that they have 127 eyes of 120 patients. You would have to have two eyes per patient in the analysis if you are carrying out a paired t-test.
3. In table 1, they present the IOP, visual acuity and CCT in two columns without presenting the average difference. It is the average difference that is being tested, not the difference in means between eyes. It is fine to show the means for each eye, but they need to also show the average difference. The layout in the table makes it appear that they have treated this as independent data. This is further unclear because they do not put N’s in the table. They need to show the number of eyes.
4. Their expression of the results for Table 1 also makes it appear that they did not analyze the average difference: “The average CCT in the affected eye was significantly higher than the unaffected contralateral control eye (p<0.0001).”

You state that “The relationship of ΔCCT with the final diagnosis of glaucoma or need for surgery/additional management was analyzed using Spearman’s correlation.” Glaucoma diagnosis and need for surgery/additional management are dichotomous variables. So, the appropriate analysis would be a t-test of ΔCCT between those in the “yes” and “no” categories of these two variables, not a Spearman correlation. You would do the same analysis here as you have just stated in the previous sentence.

Table 2 is very hard to navigate. Here is a suggestion for a clearer layout. They need to put N’s in their tables! Also, if this data is paired, the average ΔCCT should be the same as the difference between the mean in the affected eye and the normal eye. Their values of ΔCCT are very different from that. For ΔCCT, they need to report standard deviations.

| **Variable** | **Age** | **N** | **Affected Eye**  **Mean (SD)** | **Normal Eye Mean (SD)** | **Difference (ΔCCT) Mean (SD)** |
| --- | --- | --- | --- | --- | --- |
| Pars plana vitrectomy | ≤12 |  |  |  |  |
|  | 13-25 |  |  |  |  |
|  | 26-40 |  |  |  |  |
|  | >40 |  |  |  |  |
| Anterior vitrectomy | ≤12 |  |  |  |  |
|  | 13-25 |  |  |  |  |
|  | 26-40 |  |  |  |  |
|  | >40 |  |  |  |  |
