# Supplementary material for: Changes in corneal thickness after vitrectomy—Implications for glaucoma practice
Source: PLoS One. 2021 Apr 21;16(4):e0249945. doi: 10.1371/journal.pone.0249945 (PMC8059830; doi:10.1371/journal.pone.0249945)
Supplement: Attachment — Submitted filename: PLos one reoply to reviewers comments highlighted - Copy.docx [file pone.0249945.s003.docx]

To,

The Editor,

Dear Sir/Madam,

We hereby submit our revised manuscript “**Changes in corneal thickness after vitrectomy -implications for glaucoma practice**” for review for publication in your journal. We have professionally edited the manuscript for grammar and typo errors, as suggested. We would welcome any more suggestions or queries which can further improve our manuscript. We have included all suggestions made by reviewers for wider readership and easy understanding which has improved the impact of the manuscript considerably.

All the authors have contributed equally towards preparation of the manuscript and have no financial or proprietary interest in the products used in the study. We also declare that this article has not been published previously or under review with any other journal.

a) We have made available all supplemental files/data for the reader’s benefit and can make all clinical raw data available for public repository on request.

b) All data are available with the corresponding author or the institute database/IRB which can be shared after third party inclusion for specific interested readers for data sharing by the patients.

Thanking you

5. Review Comments to the Author

Please use the space provided to explain your answers to the questions above. You may also include additional comments for the author, including concerns about dual publication, research ethics, or publication ethics. (Please upload your review as an attachment if it exceeds 20,000 characters)

Reviewer #1: There is confusion about the methods of analysis stated in the methods and the presentation of the results. It is possible that the correct analysis was done, but that the results were presented in such a way to obscure that. I have attached details in a separate document along with a suggested alternative format for one of the tables. Either the presentation of the results should be revised or the methods should be restated to clarify what is being presented. They need to match.

Answer: We have provided the tables in the same way as stated in methods, Page 6-For ex- comparison of normal versus affected eye in methods with table1,2, comparison of Anterior and posterior vitrectomy eyes comparisons in table 2 and so forth. Also, we have provided both absolute CCT and ∆CCT as detailed in the methods section again. We have still changed table format as suggested while keeping details in table 2 for benefit of readers. Table 3 and Table S1 are also those that are stated in the methods section, page 6. We have made sure we have given all relevant data in the study for the benefit of readers rather than obscure any detail.

Reviewer #2: The Authors provided a study on the changes of corneal thickness after vitrectomy.
The Authors reported the changes in CCT, but to help understand the pathogenesis of these changes, it will be useful to correlate the change in CCT to the type and indications for the surgery. Since they stated that they have collected these data, I think performing this analysis could be valuable to complete their study.
Answer: We thank the reviewer for this suggestion and we agree with the reviewer that indication of surgery may be also looked at. We would like to clarify that we looked at type of vitrectomy done- anterior versus posterior rather than look at indications of surgery which may be varied and would dilute the objectives of this study (say for example surgery for dislocated Lens versus DR versus ELM peeling +PPV- there would be too many surgical variables all of which cannot be assessed statistically for each group and would never conclude with a meaningful message with several subgroup analysis). Yet, we did stratify the CCT changes by indication of surgery like PDR and TRD and did not note any significant change between the two indications and therefore have not added that in tables or text.

The manuscript is generally well written; however, I recommend an English revision for its consideration as publishable.
Answer: The manuscript has been edited by Grammarly for correcting typos and grammar, as suggested.

To follow some specific comments:

- Title: ‘Implications for glaucoma practice’. The authors did not explain how the glaucoma practice will change after their results;
Answer: The implications of a change in CCT reflecting as “raised IOP” after VR surgeries or after paediatric surgeries have been discussed in detail in two separate paragraphs in the discussion, page 9 and 10. Basically, we feel that a false alarm of glaucoma is avoided if we know that the changed CCT can falsely present as raised IOP. We thank the reviewer for ths suggestion and have added this in discussion highlighting this implication in glaucoma practice, as suggested, Page 11 and 12

.
- Methods section needs to be revised, authors need to be more precise and describe in detail the procedure they used for measuring IOP and the surgical techniques.

Answer: Being a retrospective study, we could not have blinded the trained optometrists in the study. The methods of IOP measurement and CCT are given in the methods section which have been modified as per suggestions.

Your criteria for the diagnosis of glaucoma are vague, are we talking about ocular hypertension or glaucoma? You need to be consistent and to follow specific diagnostic criteria.

Answer: We thank the reviewer for this observation. We have stated in the methods that for this retrospective study, we diagnosed secondary glaucoma in the event of a raised IOP >21mm Hg which is as per standard glaucoma diagnostic guidelines. We have also stated that disc/field changes at presentation were not mandatory for the diagnosis. So the reviewer is correct that initially they may seem as ocular hypertension; yet it is logical to understand disc/field changes would not appear immediately after raised IOP anyway. This is the reason a longitudinal prospective study may identify how many turned out be glaucoma versus ow many remained OHT wich wa not possible for this retrospective study. The entire premise of the study was to see if how this diagnostic criterion used in clinics for eyes with secondary glaucoma fares, keeping in mind the CCT changes after surgery. This mimics routine cincial scenarios and does not compare to matched /controlled prospective trials with strict definitions. The same has been discussed extensively in the results and the discussion. For a prospective study, we can revise the diagnosis based on disc/field changed over time.

- ‘standard glaucoma practice guidelines’. Which ones? Please reference.

Answer: The sentences has been rephrased, as suggested. As institutional protocol, we follow AAO and ISGEO guidelines among many others for glaucoma management.

- Stats: was the normality of the data checked before the use of t-test? If the case, please report it.
- Tables. Data reported are not easy to read, especially Table 2. Please be clearer.

Answer: Normality was indeed checked as per norms for systematic statistical analysis and parametric tests used only for normative data. Table 2 highlights the mean CCT comparisons in affected and normal eye in different age groups and between eyes that underwent anterior and posterior route of vitrectomy. This format was important to highlight that changes specific to a particular age group and particular route of surgery. This table also highlights one of the most important message of the study that is- greater ∆CCT in eyes presenting at an age <25 years, which could not have been possible had we presented differently. We have changed the tables with incorporation of all changes suggested by the reviewer. We further clarify that ∆CCT was calculated by seeing them in each eye rather than averaging CCT in affected and normal eye and seeing difference. This was because we realise that the difference between two eyes would be far too variable for each patient and also possibly by each surgery (with a lot of surgical variables). This vast variability will not be highlighted had we averaged all affected eye and all normal eyes and then seen the difference, which would have hidden and smoothened out the different variability with a single mean. This also would have been a wrong way of measuring the difference caused by each surgery or in different age groups. The number of eyes are given in table 3 clearly for each group. We have incorporated all suggestions for depicting the results in the format given by the reviewer and thank the reviewer for such a nice suggestion for explaining our results in a better way.

We also thank the reviewer for the insightful suggestion on the table format, which have been changed. We have also added number in the tables as suggested and thank the reviewer for pointing this out. We would like to clarify that there were 127 eyes of 120 patients with in eye that has absolute at presentation being excluded. Yes, the reviewer is correct that paired t test ideally requires bilateral cases only which indeed is correct and has been added in the table. Also the CCT doff therefore could be done in 120 eyes only with both eyes included only. We thank the reviewer for useful tips to highlight in the tables for a better understanding and we appreciate and have incorporated the changes.
